# 2399. Changing Demographics of Infective Endocarditis in South Carolina: An Ecological Study

**DOI:** 10.1093/ofid/ofad500.2019

**Published:** 2023-11-27

**Authors:** Phillip G Garrison, Julie Royer, Max Habicht, Sarah Battle, Kayla Antosz, Anna-Kathryn Burch, Majdi N Al-Hasan, Julie Ann Justo, Pamela Bailey

**Affiliations:** University of Alabama at Birmingham, Birmingham, Alabama; RFA, columbia, SC; Antimicrobial Stewardship Collaborative of South Carolina, Columbia, South Carolina; Prisma Health Midlands/University of South Carolina School of Medicine, Columbia, South Carolina; University of South Carolina College of Pharmacy, Columbia, South Carolina; Prisma Health Midlands/University of South Carolina School of Medicine, Columbia, South Carolina; University of South Carolina School of Medicine, Columbia, South Carolina; Dartmouth Hitchcock Medical Center; Prisma Health Richland - University of South Carolina, Columbia, South Carolina

## Abstract

**Background:**

The opioid epidemic in America is likely changing the demographics of patients with infective endocarditis (IE). Traditionally, IE is attributed to older patients and those with prosthetic heart valves or cardiac devices. Rising rates of opioid use (OU), usually via injection, has resulted in younger patients being diagnosed with IE, which raises treatment challenges.

**Methods:**

This retrospective ecological study analyzed hospitalization records from the South Carolina Revenues and Fiscal Affairs Office (SC RFA) from 2016-2021. ICD-10 codes for the diagnosis of IE yielded 7,175 hospitalizations with 5,066 unique patients. Cases of IE with concurrent OU were identified. Rurality of hospital county, hospital teaching status, hospital bed size, length of stay, total costs, discharge status, and distance to hospital zip were reviewed. Data was analyzed by year and age group (18-34, 35-49, 50-64,65-79, and 80+). Poisson regression was used to examine change in trends.

**Results:**

From 2016-2021, inpatient stays related to IE showed no significant trend for age groups 35 years and older (p > 0.19), while showing a significant positive trend in the 18-to-34 age group (p = 0.04). Of the 7,175 IE hospitalizations, 977 (13.6%) had a concurrent diagnosis of OU. The percentage of patients with concurrent IE and OU diagnosis increased from 7% to 17% of patients/year. The rate of inpatient stays related to concurrent IE and OU increased from 3.8 to 11.1 (p = 0.01) and 2.5 to 8.7 per 100,000 of SC population (p = 0.01), for the 18–34 year-old and 35-49 year old groups, respectively, while not significantly changing in patients 50 years and older over the study period (p = 0.57).

Rate Per 100,000 SC Population of Inpatient Stays Related to IE and OU by Age Group

**Figure:**
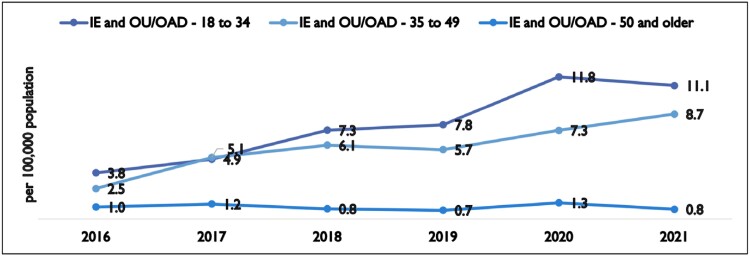

**Conclusion:**

There are few epidemiologic studies of IE since the 2015 Infectious Diseases Society of America treatment guidelines, though significant concern about the juxtaposition of the opioid epidemic with diseases like IE. There are major changes in the demographics of IE patients. This study supports the need for updated guidelines to specifically address IE in the setting of OU, particularly in younger patients. This includes possible disparities in treatment due to lack of access to ID physicians in rural communities and challenges in providing prolonged intravenous antibiotic therapy.

**Disclosures:**

**Julie Ann Justo, PharmD, MS, FIDSA, BCPS**, Gilead Sciences: Advisor/Consultant|Shionogi: Advisor/Consultant|Vaxart: Stocks/Bonds

